# Intraoperative and postoperative short-term outcomes of intracorporeal anastomosis versus extracorporeal anastomosis in laparoscopic right hemicolectomy

**DOI:** 10.3389/fonc.2023.1145579

**Published:** 2023-04-12

**Authors:** Yuhang Zhou, Yuchen Zhou, Chuandong Wang, Rong Ye, Xiaojun Lin, Song Tan, Weijie Chen, Yulong Mi, Changshun Yang, Shengtao Lin, Weihua Li

**Affiliations:** ^1^ Shengli Clinical Medical College of Fujian Medical University, Fuzhou, China; ^2^ Department of Surgical Oncology, Fujian Provincial Hospital, Fuzhou, China; ^3^ The First Affiliated Hospital of Fujian Medical University, Fuzhou, China; ^4^ Department of Colorectal Surgery, The First Affiliated Hospital of Fujian Medical University, Fuzhou, China

**Keywords:** anastomotic approach, intracorporeal anastomosis, extracorporeal anastomosis, functional end-to-end anastomosis, stapled techniques, laparoscopic right hemicolectomy

## Abstract

**Background:**

Intracorporeal anastomosis (IA) is a difficult but popular anastomotic approach for reconstruction of digestive tract after laparoscopic right hemicolectomy, which may reduce some limitations faced during extracorporeal anastomosis (EA).

**Methods:**

A retrospective review of 78 patients who underwent laparoscopic right hemicolectomy by a veteran surgeon in a high-volume public tertiary hospital, including 50 patients with IA and 28 patients with EA. The intraoperative-related factors and short-term results of the two anastomotic approaches were compared.

**Results:**

There was no significant difference in demographics and clinical characteristics between the two groups (P>0.05). The intraoperative blood loss was less (P=0.010) and the incision length was shorter (P<0.001) in the intracorporeal group. Postoperative farting time was faster (P=0.005) and postoperative pain score (VAS) was lower (P<0.001) in IA group. Although the anastomotic time of IA was shorter (P<0.001), the operative time of the two groups were similar. And number of lymph nodes harvested, NLR from POD_1_ to POD_3_, postoperative hospital stay and overall hospital stay between the two groups were comparable. Except for significant difference in abdominal infection rate, the Clavien-Dindo classification and the incidence of other postoperative complications were not statistically different. Moreover, the morbidity of abdominal infection decreased with time in the IA group (P=0.040).

**Conclusion:**

IA is a reliable and feasible procedure, which has faster anastomotic time, earlier return of bowel function and superior postoperative comfort of patient, compared to EA. The postoperative complication rate of IA is similar to that of EA, and may be improved with the IA technical maturity of surgeons, which potentially contributes to the development of ERAS.

## 1 Introduction

With the development of laparoscopic technology and staplers, the difficulty of totally laparoscopic resection and intracorporeal anastomosis (IA) is greatly reduced (1), and laparoscopic colectomy has been widely used in the treatment of colon cancer. Nonetheless, the complication rate after laparoscopic surgery is still high, which in part is related to the failure of anastomosis and reconstruction of the digestive tract (2). In addition to the anastomosis methods (side-to-side anastomosis, end-to-side anastomosis, etc.), it has been suggested that different anastomotic approaches may affect the recovery after laparoscopic right hemicolectomy in the previous studies, which can be divided into IA and extracorporeal anastomosis (EA) (3). IA is a critical step in total laparoscopic right hemicolectomy, in which all resection and anastomosis of bowel are performed intracorporeally, with a small incision to dislodge the intraoperative specimen (3); while EA refers to the laparoscopic-assisted resection and anastomosis after the externalization of the bowel through an abdominal incision (4). The theoretical advantages of laparoscopic IA include but not limit that it would avoid having to extract the bowel and mesentery through the thick abdominal wall in obese patients; it would reduce the hazard of intestinal and mesenteric twisting and tension during EA (5, 6). However, intestinal bacteria cannot be completely eliminated even by adequate preoperative bowel preparation and the bowel requires to be opened during IA, hence it is difficult to prevent the abdominal environment from bacterial contamination due to the leakage of intestinal contents and the contact of linear staplers with intestinal lumen. In addition, the technical difficulty of IA has discouraged some young doctors. Therefore, there are still some disputes about the impact of IA and EA on the perioperative period. Our hospital is a high-volume public tertiary hospital with a dedicated colorectal cancer team which has developed laparoscopic colectomy since 2013 and performed stapled anastomosis through IA or EA. This study reviewed laparoscopic right hemicolectomy through IA or EA in recent years and compared their intraoperative and postoperative outcomes.

## 2 Patients and methods

### 2.1 Patients

From September 2019 to April 2022, 78 patients who received laparoscopic right hemicolectomy were included in this study. All of these operations were performed by the same surgeon who specialized in totally laparoscopic colectomy (TLC) and open colon surgery. The operating surgeon has hundreds of cases of intracorporeal anastomosis and extracorporeal anastomosis in laparoscopic colectomy experience. The decision of anastomotic approaches was made randomly by the chief physician during the operation. The patients did not participate in the decision-making of anastomosis approaches.

**Figure 1:**
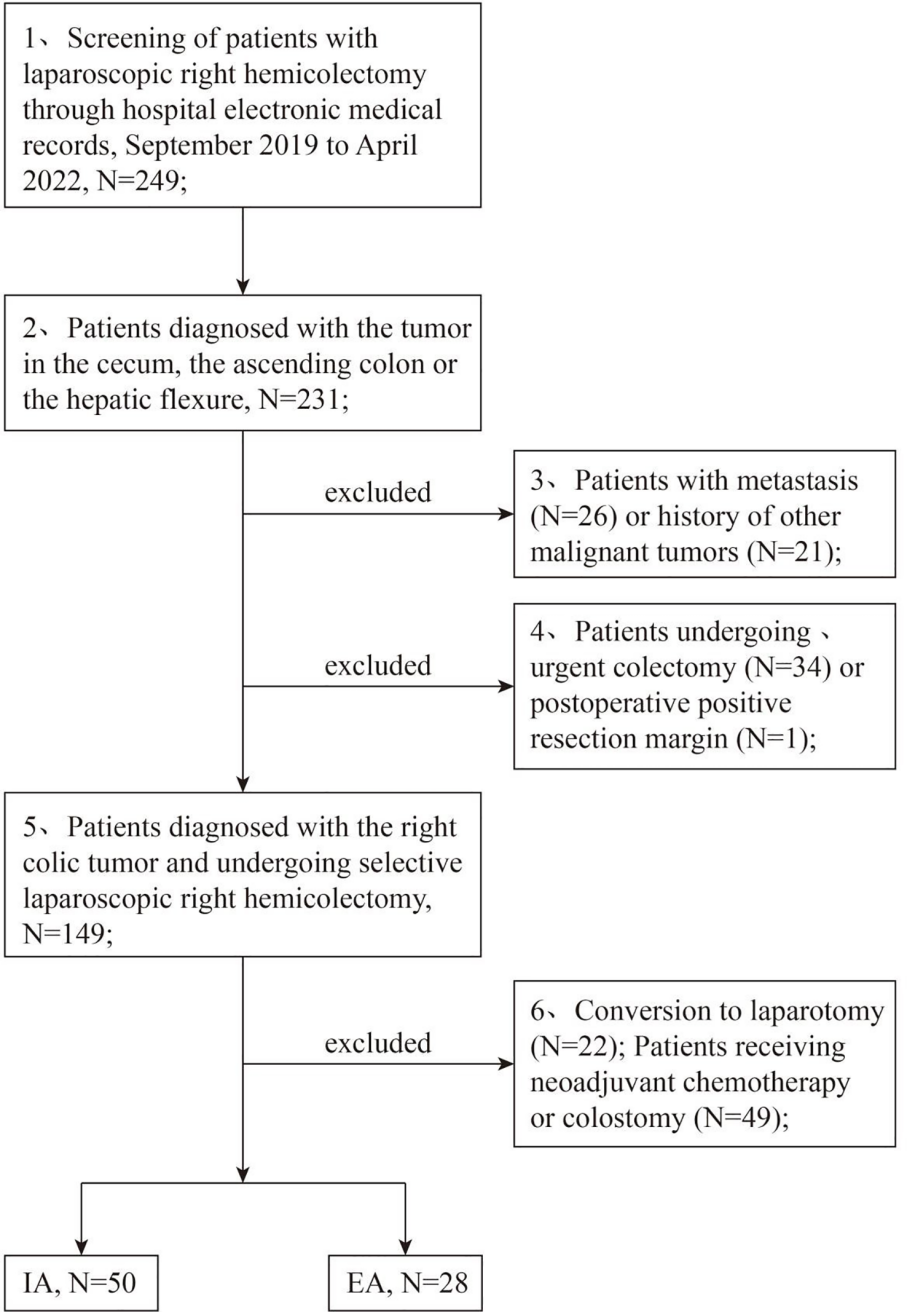
Flowchart of IA and EA Patient Selection in laparoscopic right hemicolectomy. IA, intracorporeal anastomosis; EA, extracorporeal anastomosis.

Inclusion criteria included the preoperative diagnosis of the tumor in the cecum, the ascending colon or the hepatic flexure; laparoscopic right hemicolectomy through IA or EA; postoperative negative resection margin. Patients with other malignant tumors, urgent colectomy due to complications, coagulation dysfunction or organ dysfunction, having received neoadjuvant chemotherapy or colostomy were excluded (**Figure 1**). Written informed consent was obtained from all selected patients. This study was reviewed and approved by the Ethics Committee of Fujian Provincial Hospital.

Baseline characteristics of patients included age, sex, Body Mass Index (BMI), American Society of Anesthesiologists classification (ASA), TNM staging and prior abdominal surgery. Intraoperative-related factors that included anastomotic approaches, anastomotic time, operative time, length of incision, number of harvested lymph nodes and intraoperative blood loss were recorded. Intracorporeal or extracorporeal anastomotic time started with punching in the ileal and colonic stumps and ended with closing the common opening of anastomosis, which was recorded by watching surgical video. Postoperative short-term outcomes collected included time to first passage of gas, visual analog scale (VAS) for postoperative pain, postoperative hospital stay, total hospital stay, neutrophil-to-lymphocyte ratio (NLR) during POD1 and POD3, Clavien-Dindo grade and postoperative complications. Among post-complications, postoperative ileus refers to the lack of bowel movement in the early postoperative stage (3-7 days after surgery). It was defined as the inability to fart, defecate and restore the tolerance for eating (7), then was diagnosed ultimately by imaging evidences like X-ray or CT, etc.

### 2.2 Surgical technique

Amongst 78 patients, IA was performed in 50 patients and EA was performed in 28 patients. In the intracorporeal group, ileocolic and right colic vessels were exposed and ligated laparoscopically at vascular pedicles, and the mesocolon and ileal mesentery were completely liberated before transection of colon. The cutting line was perpendicular to the colonic axis for intracorporeal resection using a linear stapler equipped with the cutting knife. After laparoscopic resection of specimen, two 5-10mm holes were made in the ileal and colonic stumps, and two prongs of linear staplers were inserted into the two holes for intracorporeal functional end-to-end anastomosis (FEEA, also known as side-to-side anastomosis). The mucosal lumen of anastomosis was checked carefully for hemostasis after 10-30 seconds of evenly clamping stapler. Then the common opening of FEEA was closed with the same stapler. The serosal bleeding of anastomosis was examined and was stanched with electric scalpel. Then the hole of Trocar at umbilical region was selected as incision to install the incision protector and remove the specimen.

In the extracorporeal group, ileocolic and right colic exteriorization and enterotomy were performed by widening the hole of Trocar at umbilical site, which followed by using linear staplers for extracorporeal FEEA and closing the common opening. The mesenteric notch was sutured after the blood supply of the anastomosis was checked, and the anastomosed bowel was placed back into the abdominal cavity.

### 2.3 Statistical analysis

All statistical analyses were conducted using SPSS software (IBM SPSS Statistics for Windows, version 25.0, Armonk, NY, USA). The independent samples t-test was used for the comparison of measurement data with normal distribution among groups. The Wilcoxon rank-sum test was used to compare the measurement data with skewed distribution and compare the ordinal data among groups. The chi-square test or Fisher’s exact test was used for the analysis of count data. P-values < 0.05 were considered statistically significant.

## 3 Results

From September 2019 to April 2022, 44 males and 34 females were included in this retrospective study with a mean age of 61.97 years (range: 40 - 88 years) and a mean BMI of 22.74 kg/m2 (range: 15.33 - 32.95 kg/m2). Among them, 28 patients underwent EA and 50 patients underwent IA. All patients are ASA II-III and survival within 30 days of surgery. There was no difference in demographics and clinical characteristics between the two groups (P>0.05) (**Table 1**).

**Table 1:** Baseline clinical characteristics of patients between the extracorporeal anastomosis (EA) group and intracorporeal anastomosis (IA) group.

	EA group (n=28)	IA group (n=50)	P value
Age	61.29 ± 15.92	62.36 ± 10.46	0.750
Sex			0.922
Male	16(57.1%)	28(56.0%)	
Female	12(42.9%)	22(44.0%)	
BMI	23.59(5.00)	22.09(4.00)	0.288
ASA classification			0.179
II	8(28.6%)	22(44.0%)	
III	20(71.4%)	28(56.0%)	
Prior abdominal surgery	4 (14.3%)	7 (14.0%)	1.000

Both groups completed laparoscopic right hemicolectomy and lymph node dissection. Intraoperative conditions of two anastomotic approaches are shown in **Table 2**. None of selected patients were converted to laparotomy. No statistical difference was observed with respect to lymph node dissection and operation time (P>0.05), yet anastomotic time between the two groups were significantly different (P<0.001); the anastomosis time of EA group was significantly longer than that of IA group. Compared with EA, the IA group had less intraoperative blood loss (median: 30 mL, P=0.010) and smaller incision (median: 3 cm, P<0.001) (**Table 2**).

**Table 2:** Intraoperative conditions of including patients.

	EA group (n=28)	IA group (n=50)	P value
Intraoperative-related factors			
Operative time(min)	200.0(84.0)	190.0(60.0)	0.136
Anastomotic time (min)	8.25 ± 1.47	6.06 ± 1.09	<0.001
Incision length(cm)	6.0(0)	3.0(2.0)	<0.001
Estimated blood loss (ml)	40.0(30.0)	30.0(0)	0.010
Lymph nodes harvested	26.0(23.0)	25.0(14.0)	0.750

EA, extracorporeal anastomosis; IA, intracorporeal anastomosis.

Postoperative outcomes of two groups are manifested in **Table 3**. No statistical difference between the two groups was found in terms of postoperative hospital stay (P=0.294), totally hospital stay (P=0.366) and NLR during POD1 and POD3 (P>0.05), but the postoperative pain scores of IA group significantly decreased (P<0.001) and the first fart time of EA group significantly delayed (P=0.006). 27 patients in the two groups had at least one postoperative complication, including anastomotic leakage, anastomotic bleeding, abdominal infection, surgical site infection and postoperative ileus, and thereof the incidence of abdominal infection between groups existed statistical difference (P=0.044), while there was no significant difference in other complications (**Table 3**).

**Table 3:** Postoperative outcomes of two groups.

	EA group (n=28)	IA group (n=50)	P value
Postoperative short-term outcomes			
NLR of POD_1_	10.54(10.07)	11.73(8.53)	0.359
NLR of POD_2_	6.91(5.79)	7.35(9.65)	0.662
NLR of POD_3_	5.54(5.79)	5.19(5.26)	0.567
first time to flatus (h)	67.6 (28.0)	48.8(43.0)	0.006
Postoperative pain (VAS)	7.0(1.0)	5.0(1.0)	<0.001
Postoperative hospital stay (day)	7.5(2.0)	8.0(4.0)	0.294
Totally hospital stay (day)	12.0(2.0)	12.0(9.0)	0.366
Clavien-Dindo classification			0.092
0	19	33	
I	5	2	
II	3	9	
III	0	5	
IV	1	1	
Overall postoperative complications	9(32.1%)	18(36.0%)	0.731
Anastomotic leakage	0	2(4.0%)	0.534
Anastomotic bleeding	0	1(2.0%)	1.000
Abdominal infection	0	9(18.0%)	0.044
Pulmonary infection	4(14.3%)	7(14.0%)	1.000
Surgical site infection	2(7.1%)	2(4.0%)	0.945
Postoperative ileus	1(3.6%)	4(8.0%)	0.776

EA, extracorporeal anastomosis; IA, intracorporeal anastomosis.

Patients in the IA group were divided into three groups based on timeline: from September 2019 to August 2020 (group A), from September 2020 to June 2021 (group B), and from July 2021 to April 2022 (group C). 6, 3, and 0 cases of abdominal infection were observed respectively among the 3 groups (P=0.040), without difference between each group (P>0.05) (**Table 4**).

**Table 4:** Abdominal infection of intracorporal anastomosis in different periods.

		Group A^a^	Group B^b^	Group C^c^	P value
Abdominal infection	No	11(64.7%)	17(85.0%)	13(100.0%)	0.040
	Yes	6(35.3%)	3(15.0%)	0(0%)	

^a^ From September 2019 to August 2020; ^b^ From September 2020 to June 2021; ^c^ From July 2021 to April 2022.

## 4 Discussion

The feasibility and safety of laparoscopic colectomy in terms of radical resection have been demonstrated by some trials with high-level evidence (8, 9). However, different types of anastomotic approaches have their own drawbacks. Theoretically, EA requires a larger abdominal incision compared to IA to achieve ileocolic exteriorization, and undue traction on the mesentery may lead to bleeding during EA (10). The result of our study demonstrated that intraoperative blood loss and incision length were reduced in IA different from EA, and a smaller incision achieved by the IA technique can decrease the hazard of incisional hernias and postoperative pain (11, 12). In our study, the postoperative pain complained basically came from the surgical incision. The incision of TLC with IA is mainly used for the removal of surgical specimens, including trans-umbilical incision which our team selected, Pfannenstiel incision and trans- McBurney incision, natural orifice. Although some studies have pointed to a lower incidence of incisional hernias using the Pfannenstiel incision compared with using the umbilical incision (13–15), none of incisional hernia was observed in our patients, so this view has not been confirmed by us yet. The umbilicus was usually employed for a Trocar and slightly enlarged to extract the specimen to avoid adding an incision. We predicted this would alleviate postoperative pain, less adhesions and offer decent cosmesis. Then our study verified this hypothesis, which tallied with the findings of Fabozzi et al. (16).

Our study showed that the first postoperative fart time of patients in IA group was faster than EA; previous studies have also found that patients receiving IA recover their diet and defecate more quicker than EA (17, 18). These evidences suggested that bowel function recovery after IA was faster, and less bowel manipulation and reduction of traction were thought to contribute to it (10, 19). In some research of IA, the bowel peristalsis recovery after overlap anastomosis (OLA) was found faster than after FEEA, which may be due to isoperistaltic pattern after OLA creating more natural digestive reconstruction over antiperistaltic pattern after FEEA (20, 21). However, these studies also showed that the difference between two anastomotic mode was not significant in the incidence of post-complications; On the other hand, the OLA is more complicated than FEEA, which will prolong the anastomotic and operative time (20–22). Our chief surgeon was adept at FEEA and we opined that the theoretical advantages of OLA may be counterbalanced by our extensive experience of FEEA.

IA reduces tissue trauma by better visualization of anastomosis and abdominal manipulation under the laparoscopic lens, which may also be one of explanations for less intraoperative bleeding or one of reasons for less postoperative inflammatory reaction. NLR is a validated prognostic scoring system, which uses two different leukocyte count components to predict short-term systemic inflammatory response; previous literatures suggested that IA had less inflammatory reaction than EA (17, 23). In contrast, there was no significant difference in NLR between the two anastomoses in our study, which was speculated to be related to the postoperative complications of the two anastomotic approaches.

Some scholars opined that EA needed more time to arrange the bowel and mesentery to avoid mesenteric torsion (10, 24). Although our study discovered that the anastomotic time of IA was shorter than EA, there was no significant difference respect to the operative time, probably because the extra time was taken in separating the mesentery and ileocolic vessels intracorporeally (25). Some studies (18, 26, 27) even found that TLC required longer operative time than laparoscopic-assisted colectomy (LAC), which may be related to the difficulty of performing IA. We conjectured that the mastery of IA and tacit understanding of the team cooperation would curtail the anastomotic time and alleviate the difficulty of IA operation. Hanna et al. (6) also found the operative time was improving steadily by analyzing the learning curve in IA, believing the overall operative time of TLC by surgeons well versed in IA should be comparable to that of LAC (5, 17). In addition, it is difficult to judge whether the bowel or mesentery twisted owing to the limitation of the assisted incision during EA; While IA reduce the time of avoiding intestinal and mesenteric twisting and tension depending on clear laparoscopic observation. Different types of sutures and materials may also impact the effect of anastomosis. It has been pointed out that the post-complications of double-layer anastomosis is less than single-layer anastomosis, and the use of barbed sutures in double-layer suturing can shorten the anastomotic time in TLC (28, 29). Although barbed sutures have not been frequently used by our team hitherto in TLC, it will be considered to utilize for further reducing the anastomotic time and post-complications of IA, which is significant for the development of totally laparoscopic surgery.

In addition to intraoperative-related factors, the occurrence of postoperative complications is often discussed when comparing the anastomotic approaches, and the reports are conflicting in the previous systematic reviews. Our research found that the incidences of postoperative complications of IA and EA were equivalent, and there was no difference between them when stratifying postoperative complications according to Clavien-Dindo classification in detail.

Theoretically it is redundant to drag out the bowel from coelom for stapling during IA, hence fewer bowel operations are required for IA and the incisions are less likely to be contaminated (30). However, no significant difference was observed in the postoperative ileus and surgical site infection (SSI) between the two anastomotic techniques, which may be induced by the low morbidity seemly due to limited sample size. Anastomotic leakage is one of the most concerned postoperative complications. It has previously been shown that exteriorization of the bowel, hand-sewn anastomosis and compromised vascular supply by lengthening of the mesentery bring about a higher incidence of anastomotic leaks in EA (17, 31). Our team was used to performing manual suture after stapled anastomosis regardless of whether IA or EA, which may be the reason for the low incidence rate and statistical similarity of anastomotic leakage and anastomotic bleeding between the two anastomotic methods in our study. It was worth noting that half of the complications in the IA were abdominal infections, which was significantly different from EA. Although many scholars indeed speculated that opening the bowel during IA increased the hazard of abdominal infection, their findings did not definitely support it (2, 17). We had found several years ago that the postoperative abdominal infection rate in IA was higher than EA, so we had tried a variety of methods to prevent it, including adequate preoperative bowel preparation, sufficient intraoperative peritoneal lavage and improving the postoperative nutrition and immunity. Further research revealed that the abdominal infection rate after IA had been descending in recent years, which may be related to the preventive measure or IA technical strides of our surgeons. We infer that with the comfort of our surgeons for IA technique, the abdominal infection after IA will further decline in the next few years (6); and as the incidence of postoperative complications such as abdominal infection decreases, the superiority of IA in postoperative recovery may be gradually revealed, including shortening the length of hospitalization and reducing the inflammatory response, which is relevant for the realization of “enhanced recovery protocol (ERAS)”.

## 5 Conclusion

In our opinion, IA is a safe and reliable technology and even has an advantage over EA in terms of anastomotic time, incision length, during surgery, intraoperative blood loss, postoperative pain and bowel function recovery. Along with the advance of IA technique, smaller hazard of postoperative complications will be obtained and the potential of IA in ERAS will be gradually realized. In addition, due to the limited patient number in this study, more cases will be required to verify these views in the future.

## Data availability statement

The original contributions presented in the study are included in the article/supplementary material. Further inquiries can be directed to the corresponding authors.

## Author contributions

YHZ, YCZ, CW and RY analyzed the data and wrote the manuscript. XL, ST and WC searched and collected data. YM and CY: modify the draft. SL, WL: designed the study. All authors contributed to the article and approved the submitted version.
